# Experimental migration upward in elevation is associated with strong selection on life history traits

**DOI:** 10.1002/ece3.5710

**Published:** 2019-10-02

**Authors:** Megan L. Peterson, Amy L. Angert, Kathleen M. Kay

**Affiliations:** ^1^ Ecology and Evolutionary Biology University of California Santa Cruz Santa Cruz California; ^2^ Department of Botany and Zoology University of British Columbia Vancouver BC Canada

**Keywords:** climate change, elasticity, life history, *Mimulus guttatus*, range shifts, selection

## Abstract

One of the strongest biological impacts of climate change has been the movement of species poleward and upward in elevation. Yet, what is not clear is the extent to which the spatial distribution of locally adapted lineages and ecologically important traits may also shift with continued climate change. Here, we take advantage of a transplant experiment mimicking up‐slope seed dispersal for a suite of ecologically diverse populations of yellow monkeyflower (*Mimulus guttatus* sensu lato) into a high‐elevation common garden during an extreme drought period in the Sierra Nevada mountains, California, USA. We use a demographic approach to quantify fitness and test for selection on life history traits in local versus lower‐elevation populations and in normal versus drought years to test the potential for up‐slope migration and phenotypic selection to alter the distribution of key life history traits in montane environments. We find that lower‐elevation populations tend to outperform local populations, confirming the potential for up‐slope migration. Although selection generally favored some local montane traits, including larger flowers and larger stem size at flowering, drought conditions tended to select for earlier flowering typical of lower‐elevation genotypes. Taken together, this suggests that monkeyflower lineages moving upward in elevation could experience selection for novel trait combinations, particularly under warmer and drier conditions that are predicted to occur with continued climate change.

## Graphical abstract

We use a high‐elevation common garden experiment to test the potential for up‐slope migration and corresponding patterns of phenotypic selection in yellow monkeyflowers. We find that lower‐elevation populations have higher fitness than local populations, but experience strong selection on life history traits in a high‐elevation environment. Lineages moving upward in elevation with continued climate change could experience selection for novel trait combinations.

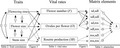

## 1 INTRODUCTION

As climate change progresses, many species are expected to respond by tracking their historical climate envelopes through space. Shifts in distribution poleward or upward in elevation have already been observed in many species (Chan, Hill, Ohlemüller, Roy, & Thomas, 2011; Lenoir, Gégout, Marquet, Ruffray, & Brisse, 2008; Moritz et al., 2008; Parmesan & Yohe, 2003). However, species are not homogeneous throughout their range, and climatic tolerances at the species level generally arise from a collection of locally adapted lineages with narrower tolerances (Kelly, Sanford, & Grosberg, 2012; Sheth & Angert, 2014). Studies that have incorporated intraspecific variation into distribution forecasts have found that species‐level range shifts will often involve the redistribution of intraspecific lineages (reviewed in Peterson, Doak, & Morris, 2019). For example, warmer‐adapted lineages are predicted to expand poleward, outperforming local lineages (Angert, Sheth, & Paul, 2011; Benito Garzón, Alía, Robson, & Zavala, 2011; Kapeller, Lexer, Geburek, Hiebl, & Schueler, 2012). Some transplant experiments have also found support for this process, with local lineages outperformed by nonlocal but warmer‐adapted lineages (Gray, Gylander, Mbogga, Chen, & Hamann, 2011; McGraw et al., 2015; Wilczek, Cooper, Korves, & Schmitt, 2014). Such a pattern indicates adaptational lag, wherein local lineages are unable to adapt sufficiently quickly to track changing climate conditions in situ.

Changes in the distribution of locally adapted lineages may also cause the redistribution of ecologically important traits. For example, the distribution of vernalization requirements and flowering time in *Arabidopsis* is predicted to shift with climate change due to differing climatic tolerances among genotypes, with larger proportions of the species' range predicted to flower earlier and without requiring vernalization (Banta et al., 2012; Marcer, Mendez‐Vigo, Alonso‐Blanco, & Pico, 2016). Such shifts in the distribution of ecologically important traits could have profound impacts on species' ecological function, including interactions with other species and local population dynamics.

Most forecasts of trait distributions under climate change assume that trait–climate relationships are static through time (Wang, Hamann, Yanchuk, O'Neill, & Aitken, 2006, Angert et al., 2011, Banta et al., 2012, Marcer et al., 2016; but see Bush et al., 2016). However, selection may favor novel traits or trait combinations during climate‐induced range shifts. For example, as lineages migrate into newly climatically suitable habitats, they may face strong selection on key traits from other aspects of the local environment. Evolutionary changes in key traits may also feed back to influence the rate or magnitude of range shifts (Banta et al., 2012; Phillips, Brown, Webb, & Shine, 2006). Studies that measure phenotypic selection during range shifts are necessary to understand how the distribution of ecologically important traits may change under forecasted climate conditions.

Life history traits, which govern the timing of and allocation of resources to different portions of the life cycle, may be under particularly strong selection in changing climate conditions. Life history shifts, including changes in the timing of reproduction, have been among the strongest and most consistent biological responses to climate change (Anderson, Inouye, McKinney, Colautti, & Mitchell‐Olds, 2012; Both et al., 2004; Parmesan, 2007). Adaptation to local climates often involves changes in life history traits, including the timing of and allocation to sexual reproduction (Colautti & Barrett, 2013; Stearns, 1992; Stinchcombe et al., 2004). Finally, there is growing evidence that climate change has already exerted strong selection on life history traits (Franks, Sim, & Weis, 2007; Nevo et al., 2012; Thomann, Imbert, Engstrand, & Cheptou, 2015).

Here, we take advantage of an experiment simulating migration upward in elevation for lineages of yellow monkeyflower (*Mimulus guttatus* sensu lato) during an extreme drought period in the Sierra Nevada mountains, USA, to test for selection on life history traits in a high‐elevation environment. In this study, we use trait and fitness data from a common garden field experiment in which annual and perennial populations across an elevation transect were transplanted into a high‐elevation montane meadow (Peterson, Kay, & Angert, 2016). Yellow monkeyflowers exhibit substantial life history variation associated with local adaptation to climate; early‐flowering annuals are adapted to fast‐drying habitats, whereas later‐flowering perennials are adapted to mesic environments (Hall & Willis, 2006; Lowry, Rockwood, & Willis, 2008; Peterson et al., 2016). In a previous analysis of this experiment, we found that lower‐elevation perennials outperformed local montane perennials, suggesting the potential for upward‐elevation shifts in this system (Peterson et al., 2016).

Here, we use a demographic approach to ask how individual‐level variation in the timing of and allocation to sexual reproduction influences fitness of different populations when transplanted to a high‐elevation site. In particular, we experimentally mimic seed dispersal of lower‐elevation populations, as well as local high‐elevation populations, into a high‐elevation montane habitat to compare the pattern of selection on key life history traits among populations and between normal versus drought conditions. We considered both direct effects of trait variation on vital rates, and hence population growth, and indirect effects through correlated traits. We predicted that traits associated with more mesic environments and higher elevations, including later flowering, larger size at first reproduction, and larger flowers, would be favored in this montane habitat. However, we also predicted that selection might be weaker or even in an opposing direction under severe drought conditions.

## 2 METHODS

### 2.1 Study system

The *M. guttatus* (DC, Phrymaceae) species complex comprises extensive variation in morphological, floral, and life history traits (Friedman, Twyford, Willis, & Blackman, 2015; Kooyers, Greenlee, Colicchio, Oh, & Blackman, 2015; Peterson, Miller, & Kay, 2015). The evolutionary relationships among the populations used in this study remain uncertain, and the most recent taxonomic treatment recognizes annuals, low‐elevation perennials, and montane perennials as separate morphological species on the basis of life history and habitat (*Mimulus micranthus*, *M. guttatus*, and *M. corallinus*, respectively; Nesom 2012). However, genomic studies suggest that genetic variation in this complex is partitioned geographically rather than according to life history divisions (Oneal, Lowry, Wright, Zhu, & Willis, 2014; Puzey & Vallejo‐Marín, 2014) and all populations used in this study are interfertile, with viable seed set between populations ranging from 15% to 133% of seed set from within‐population crosses (M. Peterson, unpublished data), in contrast to other closely related species that tend to have stronger crossing barriers with *M. guttatus* (Diaz & Macnair, 1999; Garner, Kenney, Fishman, & Sweigart, 2016; Oneal, Willis, & Franks, 2016). Thus, we refer to these taxa as ecotypes within *M. guttatus*; however, the results and interpretations in this study would be similar regardless of their taxonomic status.

In 2010, we collected 30 maternal seed families from each of 11 populations in the central Sierra Nevada and surrounding foothills (Table S1). These populations span the range of life history variation in this region, from diminutive annuals to low‐elevation and montane perennials (Figure S1). We classified populations into four life history groups associated with different elevations, local soil moisture regimes, and life history traits. First, we classified populations as either annual or perennial based on duration in the field (i.e., senescence at fruit maturity); annuals tend to occur at lower elevations (range: 293–1,693 m a.s.l.) than perennials (range: 1,371–2,066 m a.s.l.; Table S1). We further divided low‐elevation and montane perennials based on morphology and habitat. Montane perennials occur above 1,450 m elevation and spread vegetatively through belowground rhizomes, whereas low‐elevation perennials produce aboveground stolons. We also divided annual populations into fast‐cycling annuals that flower early at a small size and robust annuals that flower later at a larger size; this variation was strongly associated with local differences in soil moisture, with the fast‐cycling annuals occurring in rapidly drying seeps whereas robust annuals occur in more mesic meadows (Figure S1). These four life history groups are well separated in a principal components analysis of trait variation (Figure S2) and also differed in their relative fitness in the common garden experiment (Peterson et al., 2016). Thus, this approach allowed us to pool populations within life history groups to increase sample sizes and power for estimating selection without mixing very different distributions of traits or vital rates (Figures S3 and S4). In a preliminary analysis, we found little evidence for population × trait interactions for fitness within life history groups (3 of 51 selection gradients), and none of these interactions reflected significant differences in the direction of selection among populations.

### 2.2 Common garden experiment

We transplanted seedlings from these 11 populations into a common garden field experiment over two years (Peterson et al., 2016). The site of the common garden was a montane meadow in Stanislaus National Forest, CA (N 38.32107, W 119.91607, 2,040 m a.s.l.), with a small stream supporting a native montane perennial population (which was included as one of the 11 transplanted populations). Our experiment occurred at the start of an extreme drought period (2012–2015) with soil moisture deficits greater than those seen in the last 500–1,000 years (Belmecheri, Babst, Wahl, Stahle, & Trouet, 2015; Griffin & Anchukaitis, 2014). Due to the cumulative nature of moisture deficits, conditions in the first year (2012) were within the range of variation in the last 100 years, whereas conditions in the second year (2013) were extremely dry (Griffin & Anchukaitis, 2014), and more similar to forecasted conditions under climate change (Reich et al., 2018). Specifically, July 2012 experienced 13 mm precipitation and mean maximum temperatures of 26.1°C, whereas July 2013 experienced 0 mm precipitation and mean maximum temperatures of 29.5°C (PRISM Climate Group).

On 12 June 2012 and 26 May 2013, following snowmelt at the common garden site, we transplanted cohorts of 40 seedlings from each of the 11 populations into randomized blocks along the stream bank within the distribution of native plants (1 individual per population per block; 40 blocks per year). Experimental seedlings were derived from field‐collected seeds (30 maternal families pooled per population) germinated in the UC Santa Cruz greenhouse in 2012 and randomly sampled from among the germinants (10–30 maternal families pooled per population) in a separate seed germination field experiment at the common garden site in 2013. To prevent genetic contamination of the native population through rhizomes, pollen, or seeds, we transplanted seedlings into 4‐inch round pots (Kord, Ontario, Canada) buried within the local substrate, emasculated flowers of any experimental plants reaching the reproductive stage, and collected any maturing fruits prior to dehiscence. Emasculation is a common technique to prevent pollen contamination in transplant experiments and has been shown to not alter flower number or seed set in several plant species (e.g., Culley, 2002; Elle & Carney, 2003).

Experimental blocks were inundated with snowmelt early in each growing season, and then slowly dried as stream flow decreased throughout summer. To reduce transplant shock and mitigate the effects of pots on root development, we watered all experimental blocks to field capacity at the time of transplant and at each census. Although the use of pots and supplemental watering may have altered some aspects of the local environment, experimental plants from montane perennial populations exhibited similar phenology and drought stress as the surrounding native population (e.g., in 2013, rates of wilting and senescence were similar between experimental and native plants; M. Peterson, personal observation.). Additional details on the common garden experiment are given in Peterson et al. (2016).

#### 2.2.1 Trait measurement

We tracked the survival, phenology, and fecundity of all experimental individuals every 3–10 days from the time of transplant through the end of the growing season (the first snowfall on 12 October 2012 and the drought‐induced death of all aboveground biomass by 27 September 2013). We measured seedling size at transplant as a covariate and three life history traits relevant to the timing of and allocation to sexual reproduction: time to first flower, plant size at first flower, and flower size. We measured seedling size on the transplant date as the product of seedling diameter measured at the widest point and at a 90° angle to that point. We measured time to first flower as the number of days from transplant to the first census with at least one open flower. We measured plant size as the diameter of the primary flowering stem at the basal node and flower size as the width of the corolla at the widest point on the first census with an open flower.

### 2.3 Population models

We built stage‐structured population matrix models for each life history group and year to estimate the population growth rate (*λ*) as our fitness measure as well as the elasticities, or the proportional contributions to *λ*, of underlying vital rates. These models are described in detail in Peterson et al. (2016), which uses life table response experiments to test for local adaptation at three nested ecological scales (between annual and perennial races, between low‐elevation and montane perennials, and among montane perennial populations); we provide a brief overview here.

#### 2.3.1 Vital rates

**Figure 1:**
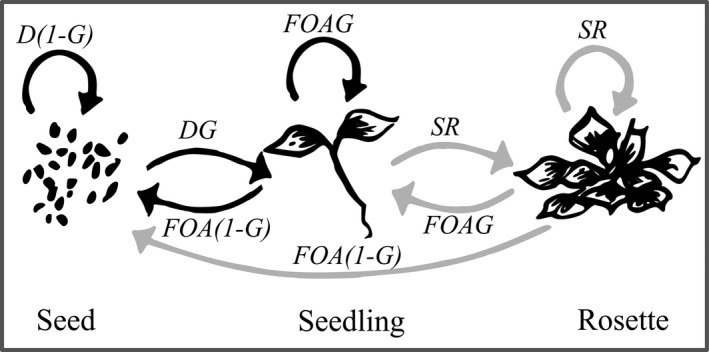
Life‐cycle graph used to construct population models for *Mimulus guttatus*. Black arrows indicate life‐stage transitions common to both annual and perennial populations, whereas gray arrows indicate transitions unique to perennial populations. At the start of the growing season, individuals can exist as seeds in the seedbank, newly germinated seedlings, or vegetative rosettes that have successfully overwintered. Letters indicate the demographic parameters that determine each annual transition rate (see text): *D* = seedbank survival rate; *G* = germination rate; 1‐*G* = dormancy rate; *F* = flower number; *O* = ovules per flower; *A* = viable seedlings per ovule; *S* = overwinter survival rate; and *R* = clonal rosettes per seedling or rosette. *A* includes a constant germination rate, whereas *G* is a population‐specific germination rate (see Section 2 for further details). We estimated *D*, *G*, and *A* as constants, but used individual‐level data for *F*, *O*, and *SR* to estimate selection (Figure 2)

We estimated yearly transition rates according to a prereproductive census at the start of the growth season following snowmelt. At this point, individuals can exist as one of three stages: seeds in the seedbank, newly germinated seedlings, or, for perennial populations, vegetative rosettes that have successfully overwintered (Figure 1). We modeled the growth of each stage‐classified life history group as ***N***(*t* + 1) = **MN**(*t*), where ***N***(*t*) is a vector of stage‐classified individuals at time *t* and **M** is a 3 × 3 matrix of transition rates parameterized according to Figure 1. For each individual and year, we estimated adult overwinter survival (*S*, defined as at least one vegetative rosette following snowmelt), clonal rosette production (*R*, the number of vegetative rosettes produced per individual following snowmelt), and flower number (*F*, the total number of flowers per individual). We recorded adult survival and counted the number of vegetative rosettes following snowmelt each year on 11 May 2013 and 1 May 2014. We also estimated ovule number per flower (*O*, the mean number of ovules per flower) by collecting second flowers from a subset of individuals per population (number of flowers per population: mean = 14, range = 3–31). We used a separate seed germination experiment to estimate a mean germination rate (*G*) for each population based on 96 field‐collected seeds left to overwinter in plug trays (98 cell plug trays; TO Plastics) at the common garden site, to account for seed overwinter survival and early viability selection (details in Peterson et al., 2016).

We also measured recruitment rates of seedlings and clonal rosettes in the native population at the common garden site to estimate a single ovule‐to‐seedling transition rate (*A* = 6.7 × 10^−4^) across all populations and years (details in Peterson et al., 2016). Since *Mimulus* produces hundreds of ovules per flower and many flowers, this approach provides a rough estimate for how ovule production scales to viable seed production. We used naturally recruiting seedlings, which incorporates germination success. However, since *A* is a constant rate for this site, we also multiply this value by a population‐specific germination rate (*G*) from a separate seed germination experiment in our population models (Figure 1). Using this method produces *λ* estimates that straddle one (Table 1), suggesting that this approach produces reasonable estimates of seed production and germination rates. However, we also compared this approach to one in which we rescaled *A* by multiplying by the relative germination rate for each population in the seed germination experiment (*G*/mean *G*) to produce population‐specific estimates of *A*. This alternative approach did not alter the elasticities of vital rates or the rank order of mean fitness (*λ*), so we chose the initial approach for consistency with Peterson et al. (2016). The seed germination and recruitment experiments are described in detail in Peterson et al. (2016). Finally, we used 0.534 as a constant yearly seed survival rate (*D*) based on a seed viability study using pooled *M. guttatus* seeds from multiple Sierra Nevada populations (Elderd & Doak, 2006).

**Table 1:** Vital rates and population growth rates for each life history group and year

Life history	Elevation (m a.s.l.)	2012					2013				
		*F*	*O*	SR	*λ*	*N*	*F*	*O*	SR	*λ*	*N*
Fast‐cycling annual	1,003 (313, 1,693)	6.96 (0.90)	229.3 (29.7)	0	0.88 (0.71, 1.07)	79	3.08 (0.36)	83.7 (9.9)	0	0.27 (0.25, 0.29)	80
Robust annual	760 (293, 1,225)	9.40 (0.88)	414.4 (32.3)	0	2.00 (1.69, 2.35)	160	5.83 (0.52)	189.5 (9.9)	0	0.68 (0.60, 0.78)	160
Low‐elevation perennial	1,443 (1,371, 1,515)	6.88 (1.23)	595.1 (43.3)	1.04 (0.36)	3.06 (2.22, 4.10)	80	4.09 (0.64)	492.2 (42.2)	0	1.09 (0.79, 1.34)	77
Montane perennial	2,024 (1,959, 2,066)	0.56 (0.13)	686.2 (84.3)	1.02 (0.33)	1.18 (0.69, 1.91)	117	0.30 (0.12)	446.7 (95.8)	0.34 (0.15)	0.47 (0.34, 0.81)	122

Note

From left to right, columns give the life history group (see Table S1), the mean (range) elevation, the mean (standard error) of each vital rate, *λ* (95% bias‐corrected confidence intervals), and the total number of individuals (pooling across populations) in 2012 and 2013, respectively. *O* gives the mean and standard error for the subset of individuals for which ovules were measured, but population models used population‐level means (see Section 2). Vital rates are abbreviated as *F* = flower number, *O* = ovule number per flower, and SR = number of rosettes after snowmelt (see Figure 1).

#### 2.3.2 Model construction

We constructed separate matrices for each life history group and year. We combined individual‐level flower number data (*F*) with population means for ovule number (*O*) and germination rate (*G*) and a constant ovule‐to‐seedling rate (*A*) to estimate individual seed and seedling production (according to Figure 1), then averaged across pooled individuals to estimate matrix transition rates. Perennial plants can reproduce either sexually by producing a new seed or seedling or clonally by producing a new rosette, but sample sizes for estimating adult performance were small and limited to 2013 when overall performance was low (due to drought; see Section 3). Rather than ignoring clonal spread through rosette production in perennial populations, we estimated vital rates (*F*, *O*, and SR) from seedlings in each year and used these values for both seedling and rosette transition rates (Figure 1). This approach assumes that rosette performance is equivalent to seedling performance within each population and year, which is likely conservative for size‐dependent transition rates. We estimated population growth rates and elasticities of matrix transition rates using the popbio package (Stubben & Milligan, 2007) in R v. 3.1.2 (R Core Development Team, 2015).

We constructed 95% bias‐corrected confidence intervals around all *λ* and elasticity estimates by resampling 10,000 bootstrap replicates. For each matrix, we resampled experimental individuals with replacement and stratified by population and year to preserve the sample sizes of the original dataset. We present the 95th percentile intervals (corrected for bias following Caswell 2001) around each estimate as an indication of statistical significance.

### 2.4 Elasticity analysis

We estimated the elasticity, or the proportional effect on population growth rate (*λ*), of each life history trait (flowering time, stem size at flowering, and flower size) using three individual vital rates—flower number (*F*), ovule number per flower (*O*), and the number of rosettes following snowmelt (SR)—and integrating their contributions to population growth (see details below).

We treated flower number (*F*) and ovule number per flower (*O*) as separate vital rates because we only collected ovule data on a subset of flowering individuals. *Mimulus guttatus* has a mixed mating system with delayed self‐fertilization in the absence of outcross pollination, and ovule number per flower is proportional to potential seed set per flower (Arathi & Kelly, 2004; van Kleunen & Ritland, 2004; Leclerc‐Potvin & Ritland, 1994). Once they had initiated flowering, individuals generally continued to produce new flowers throughout the growing season. So although we were unable to measure seed set directly, and the last few flowers produced by any individual may not have had sufficient time to mature fruits in either year, patterns of relative fitness through fruit production should be well captured by relative flower production.

**Figure 2:**
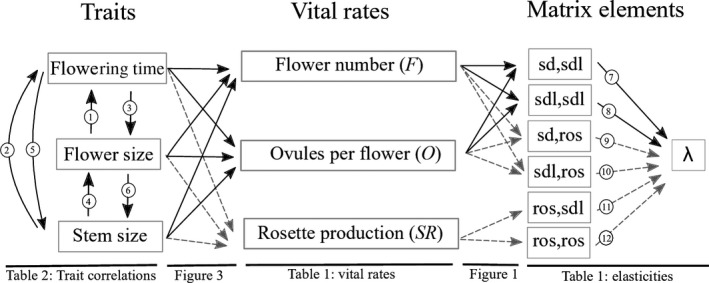
Conceptual path diagram of how trait elasticities link traits, vital rates, and matrix elements of the population model to determine the proportional effect of trait variation on population growth rate, *λ*. Arrows are numbered to indicate values given in the relevant tables (noted at bottom of figure). Matrix elements follow the format “stage*_t_*_+1_, stage*_t_*” where stages are abbreviated as sd = seed, sdl = seedling, and ros = rosette (Figure 1). Note that the sd,sd and sdl,sd transitions are not included here because they are solely determined by *D* and *G* vital rates. Black arrows indicate possible paths for either annual or perennial populations, while gray dashed arrows indicate possible paths for perennial populations only. For example, the transition from a seedling to a seed (sd,sdl) is given by FOA(1 − *G*) (Figure 1)

We estimated selection gradients through specific vital rates and trait elasticities with respect to population growth for each trait. Selection gradients measure the effect of a trait on an individual vital rate, such as flower number (Lande & Arnold, 1983). Trait elasticities incorporate selection gradients across multiple vital rates and scale them by their proportional contribution to population growth (*λ*) (Figure 2) (Van Tienderen, 2000). This is achieved by summing all paths from a trait to *λ* in an elasticity path diagram (Figure 2), where arrows represent the proportional response of a dependent variable given a proportional change in an independent variable while holding all other independent variables constant. The advantage of trait elasticities is that they incorporate selection acting through multiple vital rates as well as the fact that changes in different vital rates (e.g., fecundity vs. survival) may have very different effects on overall fitness (Horvitz, Coulson, Tuljapurkar, & Schemske, 2010). We compared trait elasticities with and without including paths through correlated traits to understand the direct and indirect effects of traits on population growth.

#### 2.4.1 Selection gradients

For each life history group and year, we estimated selection gradients as the partial regression coefficients in multiple regressions of a vital rate on all traits, including population and seedling size as explanatory variables (Lande & Arnold, 1983). We estimated mean‐standardized selection gradients (*β_μ_*) by dividing each vital rate and trait by the mean value for that life history group and year (Hereford, Hansen, & Houle, 2004). We present mean‐standardized selection gradients because they can be directly compared to elasticities, since both are in units of the trait mean (Hereford et al., 2004; Van Tienderen, 2000). We log‐transformed seedling size as log(seedling size + 0.01) prior to standardization to improve normality. Annual plants only survive for a single growing season, and all selection gradients occur through fecundity rates, including flower number (*F*) and ovule number per flower (*O*). In the montane perennial group, too few individuals flowered to fit multiple regressions with all traits. Instead, we only tested for selection gradients for flowering time and size at flowering since these were the only traits with significant or marginally significant relationships in univariate regressions (i.e., selection differentials). Flower number (*F*) and rosette production (SR) are overdispersed counts (Figure S4), so we also fit quasi‐Poisson generalized linear models of the untransformed counts with the glm package for significance testing. The same traits were significant in these models and in linear regressions of relative counts, so we present results from linear regressions here.

#### 2.4.2 Trait elasticities

For each life history group, year, and trait, we estimated its elasticity (a) based only on direct paths from a trait to *λ*, and (b) incorporating paths through correlated traits in addition to direct paths (Figure 2). For each life history group and year, we tested whether each trait was influenced by variation in any of the other traits. We estimated the proportional change in one trait given a proportional change in another trait, and holding all other traits constant, using coefficients from multiple regressions of each mean‐standardized trait on all other mean‐standardized traits (including population and log seedling size as explanatory variables). Following Van Tienderen (2000), we will refer to these coefficients as “trait correlations” although they are in fact partial regression coefficients and differ depending on the direction of trait relationships (Figure 2). One assumption of the trait elasticity approach is that the relationships among traits, vital rates, and population growth are linear (Van Tienderen, 2000). Thus, as in previous implementations of this method, we do not consider quadratic terms in our regression analyses (Coulson, Kruuk, Tavecchia, Pemberton, & Clutton‐Brock, 2003; Horvitz et al., 2010). However, in a preliminary analysis, we detected little evidence for stabilizing selection within life history groups (only 1 of 51 selection gradients). All analyses were performed in R v. 3.1.2 (R Core Development Team, 2015).

## 3 RESULTS

### 3.1 Variation in fitness among life history groups and years

Low‐elevation perennials had the highest mean fitness (estimated as population growth rate, *λ*) in a mesic montane environment, followed by robust annuals and montane perennials; fast‐cycling annuals had the lowest fitness (Table 1). 2013 was a severe drought year in the Sierra Nevada, and all components of fitness were consistently lower in this year compared to 2012 (Table 1). However, the rank order of mean fitness among life history groups did not change. Drought conditions in August and September of 2013 resulted in the senescence of all aboveground tissue, reducing the length of the flowering season for all populations and preventing any adult survival or clonal spread in low‐elevation perennials, which overwinter as aboveground rosettes. Thus, low‐elevation perennials exhibited an annual life history in 2013 in which lifetime fitness was determined by first‐year reproduction, whereas some montane perennials were able to regenerate from belowground rhizomes the following spring (Table 1).

### 3.2 Vital rate elasticities among life history groups and years

Environmental differences between years and life history differences among populations were reflected in the relative importance of different vital rates for population growth (Table 2). In annuals, the elasticity of transitions to seeds or seedlings, which involve ovule and flower production, was higher in 2012 than in 2013, when seed‐to‐seed transitions involving seedbank survival (*D*) and dormancy (1 − *G*) had greater effects on population growth due to poor seedling performance. Further, transitions to rosettes had a higher proportional effect on *λ* in montane perennials relative to low‐elevation perennials, reflecting differences in the relative importance of sexual versus vegetative reproduction among perennials. In general, low survival and fecundity in 2013 resulted in higher elasticities for transitions to more protected life stages, including seeds and belowground rhizomes in montane perennials.

**Table 2:** Elasticities of population growth to relevant transition rates for each life history group and year

Transition rate	Arrow	Fast‐cycling annual		Robust annual		Low‐elevation perennial		Montane perennial	
		2012	2013	2012	2013	2012	2013	2012	2013
sd,sdl	7	0.15 (0.12, 0.17)	0.23 (0.23, 0.24)	0.06 (0.05, 0.07)	0.16 (0.14, 0.18)	0.02 (0.01, 0.05)	0.14 (0.11, 0.18)	0.00 (0.00, 0.03)	0.03 (0.00, 0.11)
sdl,sdl	8	0.68 (0.60, 0.73)	0.22 (0.17, 0.27)	0.87 (0.84, 0.89)	0.64 (0.60, 0.68)	0.40 (0.22, 0.60)	0.69 (0.60, 0.76)	0.01 (0.00, 0.05)	0.02 (0.00, 0.08)
sd,ros	9					0.01 (0.01, 0.02)		0.03 (0.01, 0.07)	0.08 (0.05, 0.09)
sdl,ros	10					0.20 (0.13, 0.23)		0.09 (0.05, 0.12)	0.06 (0.02, 0.11)
ros,sdl	11					0.22 (0.14, 0.24)		0.11 (0.06, 0.18)	0.14 (0.10, 0.19)
ros,ros	12					0.11 (0.03, 0.26)		0.72 (0.39, 0.87)	0.38 (0.03, 0.84)
sdl,sd		0.15 (0.12, 0.17)	0.23 (0.23, 0.24)	0.06 (0.05, 0.07)	0.16 (0.14, 0.18)	0.04 (0.02, 0.06)	0.14 (0.11, 0.17)	0.03 (0.01, 0.11)	0.11 (0.03, 0.18)
sd,sd		0.03 (0.02, 0.05)	0.32 (0.27, 0.38)	0.01 (0.00, 0.01)	0.04 (0.03, 0.05)	0.00 (0.00, 0.01)	0.03 (0.02, 0.05)	0.01 (0.00, 0.07)	0.17 (0.01, 0.60)

Note

Transition rates are given as stage*_t_*_+1_, stage*_t_* with 95% bias‐corrected confidence intervals in parentheses. Arrows 7–12 refer to arrows in Figure 2; note that the sd,sd and sdl,sd transitions are not included in Figure 2 because they are solely determined by *D* and *G* vital rates. Stages are abbreviated as sd = seed, sdl = seedling, and ros = rosette. Elasticities of unobserved transitions are equal to 0 and not shown here. Note that low‐elevation perennials had no adult survival and were functionally annual in 2013.

### 3.3 Trait expression among life history groups and years

Patterns of trait expression in the high‐elevation common garden were broadly consistent with genetically based life history divergence among populations (Table 3). Fast‐cycling annual populations, which are associated with the fastest‐drying habitats, consistently flowered earliest, at the smallest size, and produced the smallest flowers. In general, perennials flowered 10–21 days later than robust annuals on average and also produced larger flowers. Among perennials, montane plants flowered later and produced larger flowers than low‐elevation perennials in 2012 but not in 2013. Somewhat surprisingly, montane perennials flowered at a smaller size than low‐elevation perennials in either year or robust annuals in 2013. Drought conditions in 2013 caused flowering to occur 2–8 days earlier than in 2012 and also reduced the size of both flowers and stems, especially for annuals.

**Table 3:** Trait means, standard deviations, and correlations with other traits

Year	Life history	Trait	Mean (*SD*)	Arrow	Correlated trait	Coefficient	Arrow	Correlated trait	Coefficient	*N*
2012	Fast‐cycling	FT	27.5 (11.88)	1	FS	−0.308	2	SS	**0.312**	56
	Annual	FS	16.25 (3.30)	3	FT	−0.183	4	SS	**0.272**	
		SS	1.75 (0.63)	5	FT	**0.591**	6	FS	**0.872**	
	Robust	FT	37.47 (15.34)	1	FS	−0.100	2	SS	**0.205**	100
	Annual	FS	16.46 (4.05	3	FT	−0.055	4	SS	−0.032	
		SS	2.41 (0.80)	5	FT	**0.261**	6	FS	−0.074	
	Low‐elevation	FT	47.49 (11.47)	1	FS	−0.129	2	SS	−0.093	30
	Perennial	FS	16.99 (3.13)	3	FT	−0.198	4	SS	0.147	
		SS	2.87 (0.75)	5	FT	−0.247	6	FS	0.255	
	Montane	FT	53 (14.37)	1	FS	NA	2	SS	−0.026	21
	Perennial	FS	19.37 (5.56)	3	FT	NA	4	SS	NA	
		SS	1.76 (0.45)	5	FT	−0.074	6	FS	NA	
2013	Fast‐cycling	FT	19.97 (10.79)	1	FS	0.045	2	SS	**0.436**	40
	Annual	FS	11.69 (3.24)	3	FT	0.013	4	SS	**0.251**	
		SS	0.75 (0.33)	5	FT	**0.318**	6	FS	**0.631**	
	Robust	FT	34.37 (13.88)	1	FS	−0.005	2	SS	0.163	96
	Annual	FS	12.72 (3.16)	3	FT	−0.002	4	SS	**0.343**	
		SS	1.17 (0.52)	5	FT	0.175	6	FS	**0.741**	
	Low‐elevation	FT	55.67 (11.07)	1	FS	0.210	2	SS	0.059	30
	Perennial	FS	16.59 (4.56)	3	FT	0.330	4	SS	0.200	
		SS	2.43 (0.89)	5	FT	0.230	6	FS	0.500	
	Montane	FT	51.15 (18.27)	1	FS	NA	2	SS	0.225	13
	Perennial	FS	15.99 (6.40)	3	FT	NA	4	SS	NA	
		SS	1.69 (0.71)	5	FT	0.335	6	FS	NA	

Note

For each year and life history group, values are the mean (sd) of each trait (FT = flowering time [days]; FS = flower size [mm]; and SS = stem size [mm]), and the coefficients for the arrows in Figure 2 (numbers 1–6). Coefficients are from multiple regressions of each mean‐standardized trait on all other mean‐standardized traits (including log seedling size and population as covariates) and give the proportional change in a trait given a proportional change in a correlated trait and holding all other trait values constant. *N* gives the sample sizes for each regression model, which only included individuals that flowered (and could be measured for each trait). Due to the limited number of flowering montane perennials, we fit univariate regressions of FT on SS and vice versa (see Section 2). Significant values (*p* < .05) are in bold.

### 3.4 Selection on life history traits

#### 3.4.1 Selection gradients

**Figure 3:**
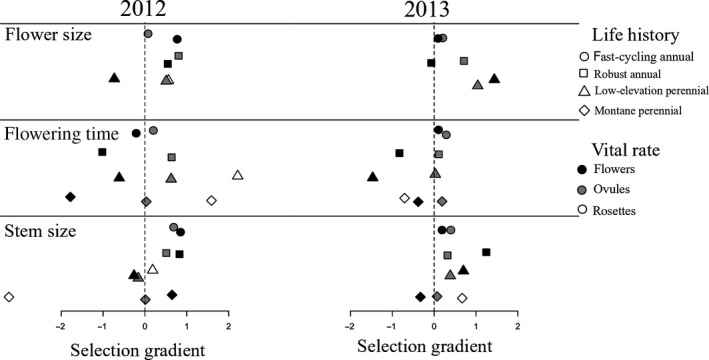
Selection gradients through particular vital rates for each trait, scaled by the vital rate elasticities, for each life history group and year. Positive selection gradients indicate that vital rates increase with larger or later trait values, whereas negative selection gradients indicate that vital rates increase with smaller or earlier trait values. Life history groups: fast‐cycling annuals (circles), robust annuals (squares), low‐elevation perennials (triangles), and montane perennials (diamonds). All values are given in Appendix S1

**Figure 4:**
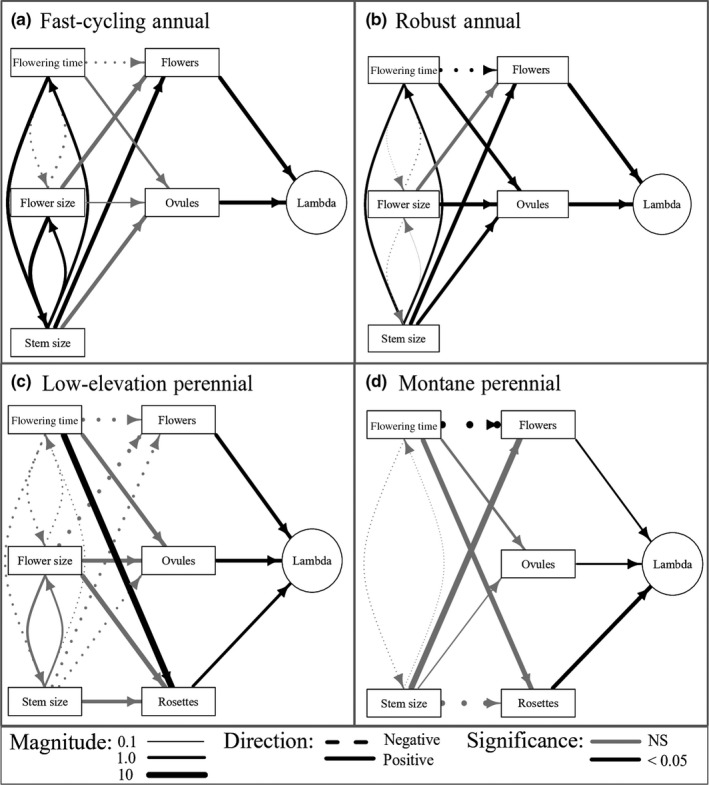
Diagrams indicating paths linking a trait to the population growth rate *λ* for each life history group in 2012. Arrow width indicates the magnitude of a relationship; dashed lines = negative relationship; solid lines = positive relationship. Positive selection gradients indicate that vital rates increase with larger or later trait values, whereas negative selection gradients indicate that vital rates increase with smaller or earlier trait values. Only black arrows are statistically significant

**Figure 5:**
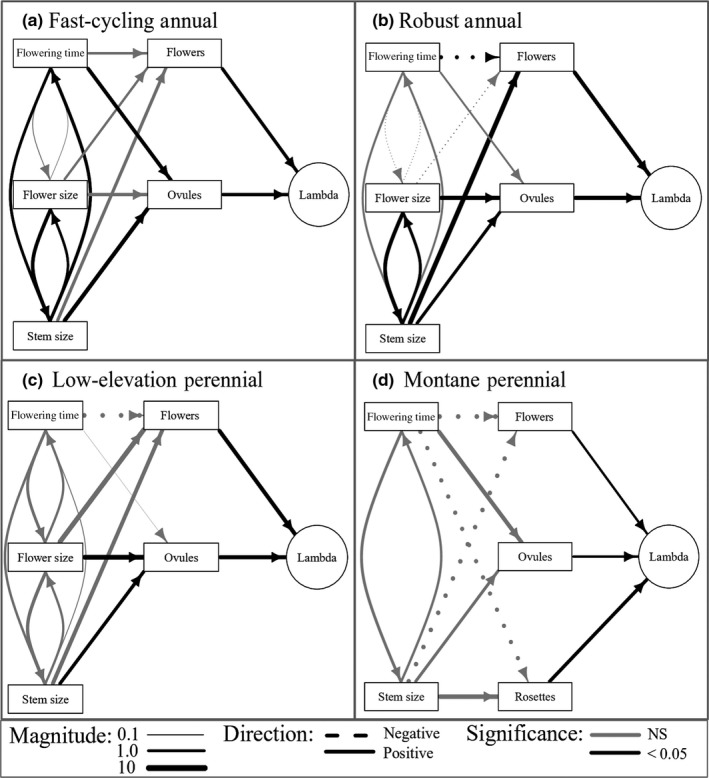
Diagrams indicating paths linking a trait to the population growth rate *λ* for each life history group in 2013. Arrow width indicates the magnitude of a relationship; dashed lines = negative relationship; solid lines = positive relationship. Positive selection gradients indicate that vital rates increase with larger or later trait values, whereas negative selection gradients indicate that vital rates increase with smaller or earlier trait values. Only black arrows are statistically significant

Selection gradients in this high‐elevation common garden varied among vital rates and years, but tended to favor larger flowers and larger stem size at flowering in both annual and perennial life history groups (Figure 3, Appendix S1). In general, selection gradients were positive for flower size, especially through ovule number (Figure 3). Statistically significant selection gradients for flower size were also always positive (Figures 4 and 5). Gradients for stem size at flowering also tended to be positive, with no statistically significant negative relationships (Figures 4 and 5). However, montane perennial plants did show a strongly negative, although nonsignificant, relationship between stem size at flowering and rosette production in 2012 (Figures 3 and 4).

Selection gradients for flowering time differed strongly among vital rates. Later flowering tended to decrease flower number but increase ovule number per flower (Figure 3). In perennial populations, later flowering was associated with increased rosette production in 2012 and decreased rosette production in 2013, though gradients were only statistically significant in 2012 (Figures 4 and 5). Statistically significant gradients for flowering time were both positive and negative (Figures 4 and 5).

#### 3.4.2 Trait elasticities

**Figure 6:**
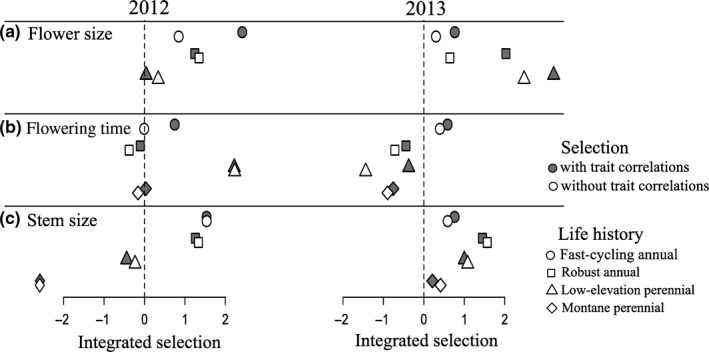
Trait elasticities for each life history group and year. Estimates are the sum of all paths (i.e., arrows in Figures 3 and 4) from a trait directly to fitness (white points) and all paths including those through correlated traits (gray points). Positive elasticities indicate that *λ* increases with larger or later trait values, whereas negative elasticities indicate that *λ* increases with smaller or earlier trait values. Life history groups: fast‐cycling annuals (circles), robust annuals (squares), low‐elevation perennials (triangles), and montane perennials (diamonds)

By integrating selection gradients across multiple vital rates, trait elasticities represent the net pattern of selection for each trait. We found that larger flowers were consistently favored across all populations and years (Figure 6a), and larger size at flowering was consistently favored in all annual populations (Figure 6c). Further, the earliest‐flowering populations, fast‐cycling annuals, experienced consistent selection for later flowering time (Figure 6b). Perennial populations, however, experienced weak or fluctuating selection on flowering time and size at first flower between years. In 2012, perennial populations experienced selection for later flowering and smaller stem size at flowering, whereas in 2013, selection favored earlier flowering and larger stem size at flowering (Figure 6b,c).

Indirect selection through correlated traits tended to either have small effects on trait elasticities or magnify their effects by acting in the same direction (Figure 6). Trait correlations among flowering time, flower size, and stem size at flowering tended to be positive, with only positive correlations statistically significant (Table 2), suggesting trade‐offs within life history groups between early reproduction and the biomass allocated to reproduction. Positive trait correlations increased the strength of selection in some cases. For example, correlations between flower size and stem size tended to be positive (Table 2), increasing selection for larger flowers through indirect selection for larger stem size (Figure 6a). Only in one case did indirect selection strongly oppose direct selection (Figure 6b); direct selection for earlier flowering in low‐elevation perennials in 2013 was opposed by strong indirect selection for larger flowers and stems at first flower (Figure 5c), though these paths were not statistically significant.

## 4 DISCUSSION

We used a demographic approach to explore how selection acts on life history traits through specific vital rates and also net population growth in annual and perennial monkeyflowers when experimentally transplanted upward in elevation into a montane site. We found that selection generally favored some perennial traits, including larger flowers and larger stem size at flowering, but that drought conditions selected for earlier flowering in all but the earliest‐flowering fast‐cycling annuals. Together, this suggests that upwardly migrating lineages in this system could experience selection for novel trait combinations in the warmer and drier conditions forecasted to occur with continued climate change (Reich et al., 2018).

By experimentally transplanting lower‐elevation populations into a montane site, we found evidence of adaptational lag, as the local montane populations had lower fitness than most lower‐elevation populations in both years. In a previous analysis of this data, perennial populations together were found to outperform annual populations, suggesting local adaptation of a perennial life history strategy (Peterson et al., 2016). However, by separating annual populations from the fastest‐drying seep habitats from those from more mesic meadows (Figure S1), we found that only the most ecologically divergent fast‐cycling annuals had lower fitness than the local montane populations. This suggests that lower‐elevation populations may be able to successfully establish in montane habitats, but that this will be mediated by similarity in local soil moisture regimes. Other transplant studies have highlighted the role of broad temperature gradients in mediating adaptational lag (Wilczek et al., 2014).

Patterns of phenotypic selection highlight the role of soil moisture, both among life history groups from differing soil moisture regimes and between normal versus drought years during the experiment. Throughout the species range, perennial monkeyflowers in more mesic environments tend to flower later and have larger flowers and stems (Friedman et al., 2015). We found a similar pattern in this study, with later flowering, larger flowers, and larger stems both in populations from more mesic environments and also in 2012, which was within the normal range of soil moisture for this site. Interestingly, selection consistently favored larger flowers, whereas the direction of selection on flowering time and stem size differed among populations and years. Later flowering was favored under normal conditions, whereas earlier flowering was favored in an extreme drought year; only the earliest‐flowering annuals continued to experience selection for later flowering under drought conditions. Under normal conditions, larger stems were favored in annuals and smaller stems in perennials, whereas larger stems were always favored in a drought year. This suggests a potential trade‐off between allocation to sexual versus vegetative reproduction, with drier conditions favoring greater investment in sexual reproduction. Warmer and drier conditions, such as occurred in 2013, appear to select for a combination of traits currently associated with higher‐elevation and more mesic‐adapted populations (e.g., larger flowers and size at flowering) and traits currently associated with lower‐elevation and more drought‐adapted populations (e.g., earlier flowering). Although the severe drought was the most obvious environmental difference between the two years in this study, this was a product of changes in both temperature and precipitation that could have affected vital rates in different ways. Identifying the specific environmental variables that drive patterns of selection is difficult, and experimental manipulations of climate are necessary to confirm these patterns since other environmental factors may also have differed between years in our study.

The potential for populations to respond to selection for new trait combinations during climate‐induced range shifts is an open question. Previous work has shown that genetic correlations can strongly oppose selection for combinations of traits under climate change, even when there is sufficient genetic variation for individual traits (Chevin, 2013; Etterson & Shaw, 2001). In yellow monkeyflowers, many life history traits have been shown to be genetically correlated in ways that would oppose the pattern of selection found here, including positive correlations between larger flowers and later flowering (Kelly & Mojica, 2011; Mojica & Kelly, 2010) and between later flowering and greater vegetative growth (Friedman et al., 2015). Yet the impacts of such correlations depend crucially on their underlying genetic structure; if genetic correlations are due to linkage disequilibrium, they may be broken up by recombination or gene flow during upward migration, whereas correlations due to pleiotropy will pose longer‐term evolutionary constraints (Lande, 1979).

In this study, we used a demographic approach, which allowed us to identify and integrate the multiple paths by which traits and environmental conditions can influence fitness. Changing environmental conditions can influence not only the relationships between traits and a particular vital rate, but also the relative contributions of vital rates to population growth. For example, selection on the timing of birth in red deer was found to act through both juvenile survival and adult reproduction, with both the direction of selection gradients and the relative importance of these vital rates fluctuating among years depending on winter climate conditions (Coulson et al., 2003; Horvitz et al., 2010). In this study, we found that the direction of selection gradients for flowering time differed among vital rates, with later flowering increasing ovule number and rosette production but decreasing flower number. Further, drought conditions tended to decrease the elasticities of fecundity rates and increase the elasticities of rates related to more protected life stages, like seeds and rhizomes; this had the net result of weakening the contribution of fecundity selection to population growth and thus overall fitness. Trait elasticities integrate the effects of a trait on all vital rates, given the effects of each vital rate on *λ*, to estimate the net proportional effect of a trait on *λ*. This integrative approach often alters qualitative conclusions relative to individual selection gradients (Coulson et al., 2003; Ehrlén & Münzbergová, 2009; Gamelon et al., 2011; Horvitz et al., 2010).

We detected some very large trait elasticities (e.g., >|2|), particularly for perennials. Hereford et al. (2004) noted that large mean‐standardized selection gradients could occur for threshold traits, for which fitness increases steeply with a small change in mean trait value. Horvitz et al. (2010) documented large trait elasticities during some life stages or years (e.g., up to |6.9|), but found that trait elasticities were smaller when summed over more years and/or life stages. In this study, we tracked perennial fitness through rosette production and compared selection over two years, but this is still an incomplete understanding of perennial fitness, particularly under fluctuating environmental conditions. It is likely that a more comprehensive understanding of perennial fitness over longer time frames and a greater range of environmental conditions would uncover additional trade‐offs and fluctuating selection, resulting in smaller estimates of trait elasticities. In particular, we caution that the results in this study are based on only two annual transitions spanning quite different climate conditions. Changing environmental conditions can strongly affect vital rates in a given year, and demographic models parameterized over shorter time periods or a limited range of environmental conditions can fail to predict longer‐term population dynamics (Crone et al., 2013; Pfeifer, Wiegand, Heinrich, & Jetschke, 2006; Tenhumberg, Crone, Ramula, & Tyre, 2018). Thus, although the annual growth rates identified by this study are useful for uncovering relationships between traits and fitness under differing environmental conditions, the longer‐term demographic and evolutionary trajectories of populations depend on the full distribution of future environmental conditions and corresponding stochastic population growth rates.

Although many species have undergone poleward or upward range shifts in response to climate change, we have little understanding of how these species‐level patterns reflect the redistribution of locally adapted lineages and ecologically important traits. In particular, climate change may alter the distribution of ecologically important traits through multiple mechanisms, including phenotypic plasticity, evolutionary responses to shifting selection pressures, and migration of locally adapted lineages. By experimentally mimicking upward seed dispersal across a range of populations, we find evidence for adaptational lag coupled with strong selection on life history traits mediated by variation in local soil moisture regimes. Taken together, this suggests that low‐elevation populations in this system may be able to migrate upward in elevation as long as the local soil moisture regimes are similar, but will experience selection for more high‐elevation trait values, potentially resulting in novel trait combinations. Whether montane lineages in this system will be able to migrate beyond the current upper elevation limit—and the extent to which selection there may favor unique trait values—is as yet unknown, although the greater allocation to vegetative over sexual reproduction could limit both the dispersal capacity and evolutionary potential of montane perennials. Given the prevalence of local adaptation (Bocedi et al., 2013; Hereford, 2009; King, McKeown, Smale, & Moore, 2017; Savolainen, Pyhäjärvi, & Knürr, 2007), we expect that many species will exhibit intraspecific variation in traits relevant to climate‐induced range shifts. However, coupled transplant and selection experiments such as this one that mimic movement within and beyond current range limits are needed across a wider range of species to understand how intraspecific lineages and ecologically important traits will shift under changing climate more generally.

## CONFLICT OF INTEREST

The authors have no competing interests to declare.

## AUTHOR CONTRIBUTIONS

MLP, KMK, and ALA designed the study. MLP collected and analyzed the data. MLP wrote the first draft of the manuscript, and all authors contributed to revisions.

## Data Availability

All data are available at Dryad https://doi.org/10.5061/dryad.0716vc7.
