# Supplementary material for: Experimental migration upward in elevation is associated with strong selection on life history traits
Source: Ecol Evol. 2019 Oct 2;10(2):612–25. doi: 10.1002/ece3.5710 (PMC6988539; doi:10.1002/ece3.5710)
Supplement: Supplementary file 1 [file ECE3-10-612-s001.docx]

**SUPPLEMENTAL FIGURES**


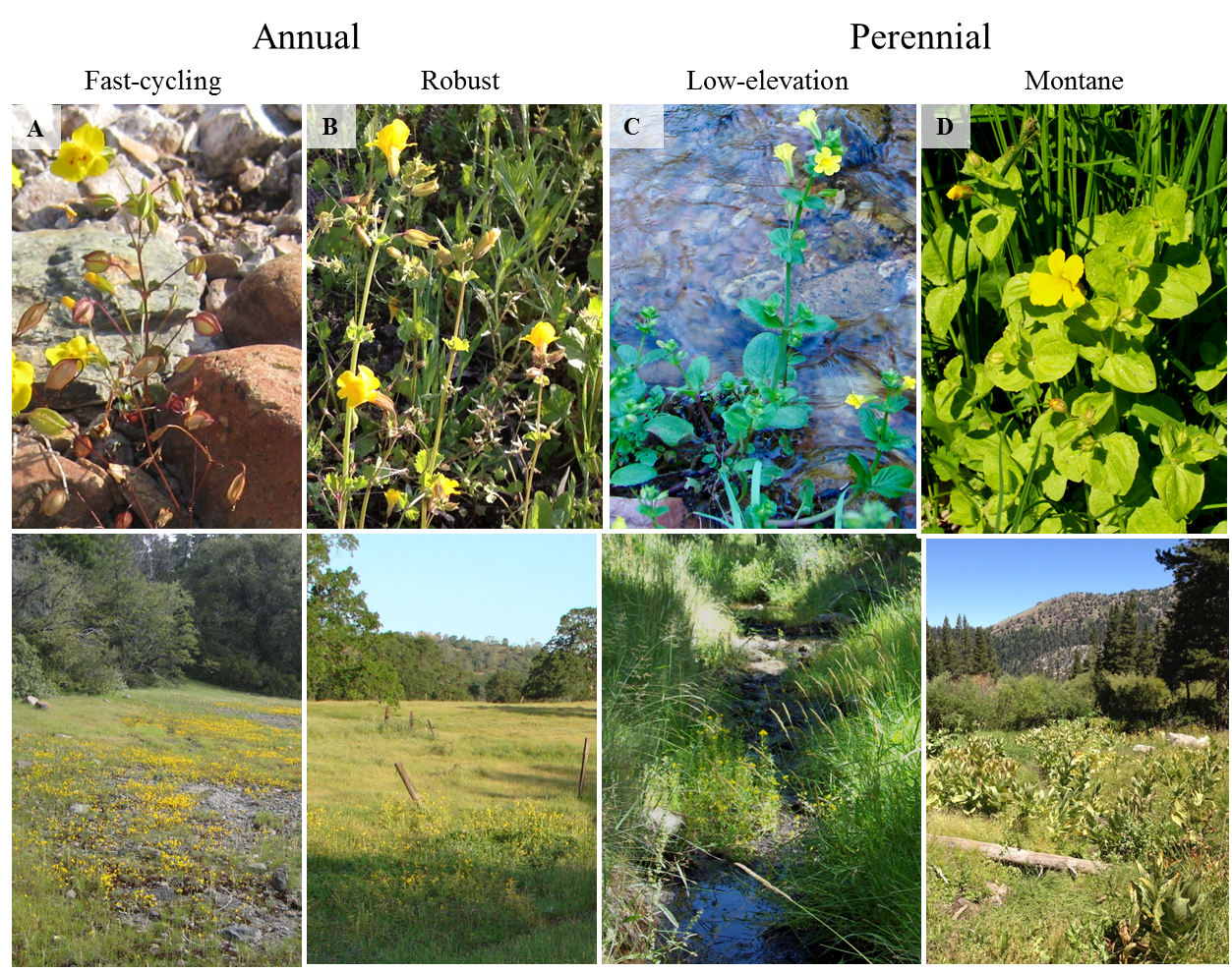


Figure S1: Characteristic growth form (top) and habitat (bottom) of the populations used in this study. A, B) Annuals occupy seasonally drying seeps or meadows and exhibit variation in the timing and size at flowering associated with variation in the length of the growth season. C, D) Perennials occupy more mesic stream banks or meadows and survive and reproduce over multiple years. Low-elevation populations (C) overwinter as above-ground rosettes connected by stolons whereas montane populations (D) produce few flowers and overwinter as below-ground rhizomes.

Figure S2: Principal components analysis of life history characteristics for 11 populations of *Mimulus guttatus*. Populations used in this study are well-separated by life history group (square = fast-cycling annual, N=2; circles = robust annual, N=4; triangles = low-elevation perennials, N=2; and diamonds = montane perennials, N=3). The black diamond indicates the population that is local to the common garden site. Axes are the first two principle components (explaining 51.9% and 24.5% of the total variance, respectively). Arrows show loadings of life history characteristics measured in each year of the common garden experiment (FS = flower size, FT = flowering time, ST = stem size, and RO = rosette production in 2012 and 2013).

Figure S3: Distributions of life history traits. Panels show the empirical probability density of flowering time (left), flower size (middle), and stem size (right) for each life history group in 2012 (top) and 2013 (bottom). Black points show the median value for each life history group and year.

Figure S4: Distributions of vital rates. Panels show the empirical probability density of flower number (*F*, left), ovule number (*O*, middle), and rosette number (*SR*, right) for each life history group in 2012 (top) and 2013 (bottom). Black points show the median value for each life history group and year.

**SUPPLEMENTAL TABLES**

Table S1: Locality and life history information for all populations in this study.

| Population | Elevation (masl) | Latitude | Longitude | Habitat | Life history group |
| --- | --- | --- | --- | --- | --- |
| Red Hills | 313 | 37.857 | -120.457 | Serpentine seep | Fast-cycling annual |
| Bald Mountain | 1693 | 38.137 | -120.094 | Gravelly seep | Fast-cycling annual |
| Peoria Basin | 293 | 37.933 | -120.520 | Creek in grassland | Robust annual |
| Traverse Creek | 681 | 38.873 | -120.818 | Serpentine creek | Robust annual |
| Sprague Rd | 842 | 37.819 | -120.152 | Creek in mixed woodland | Robust annual |
| Big Oak Flat | 1225 | 37.829 | -119.958 | Marshy meadow | Robust annual |
| Kyburz | 1371 | 38.768 | -120.290 | Stream in pine forest | Low-elevation perennial |
| Girard Creek | 1515 | 38.731 | -120.240 | Stream in pine forest | Low-elevation perennial |
| Silver Fork | 1959 | 38.664 | -120.219 | Stream banks and surrounding meadow | Montane perennial |
| Eagle Meadows | 2046 | 38.320 | -119.920 | Small stream | Montane perennial |
| Silver Creek | 2066 | 38.588 | -119.786 | Marshy meadow | Montane perennial |
